# Supplementary material for: Dissection of the in vitro developmental program of Hammondia hammondi reveals a link between stress sensitivity and life cycle flexibility in Toxoplasma gondii
Source: eLife. 2018 May 22;7:e36491. doi: 10.7554/eLife.36491 (PMC5963921; doi:10.7554/eLife.36491)
Supplement: Supplementary file 3. — File containing all of the primers used in this study, the purpose of the primer, and the Toxodb.org gene ID for each gene queried. [file elife-36491-supp3.docx]

**Supplementary File 2: Primer sequences**

| Gene | Toxodb.org ID | Purpose | 5' sequence | 3' sequence |
| --- | --- | --- | --- | --- |
| DENSE GRANULE PROTEIN 1 (GRA1) | HHA_270250 | qpcr control gene | GAGGAGGTGATGGAGACTATGA | CTCTACTGTCTCGCCTTTGTTC |
| DENSE GRANULE PROTEIN 1 (GRA1) | TGVEG_270250 | qpcr control gene | TTAACGTGGAGGAGGTGATTG | TCCTCTACTGTTTCGCCTTTG |
| SAG-RELATED SEQUENCE SRS27B | HHA_258810 | qpcr | ACAATACCCACCATAACGACAG | AAGGCTGCCATACACAAGAG |
| RHOPTRY PROTEIN ROP18 | HHA_205250 | qpcr | GACGGCCTCTTGGACTATTTAC | TCCGTTTCTTAGGCTGTTGATT |
| WD DOMAIN, G-BETA REPEAT-CONTAINING PROTEIN | HHA_219450 | qpcr | ACACTCTACCCGAGGAATACA | AGATTCCGCCTCCCTTATCT |
| HYPOTHETICAL PROTEIN_1 | HHA_253150 | qpcr | ATTGGCACTCAGGGTCTTTC | CCTGCCTTCTTGAGCTTCTT |
| PUTATIVE TRANSMEMBRANE PROTEIN | HHA_215328 | qpcr | CTCTGCAAACGCGCATATTT | AGAAATCCAGCTGTGGTATGG |
| HYPOTHETICAL PROTEIN_3 | HHA_278680 | qpcr | CACACAGCGCCTAGATAGTT | GGGTGATGTGGCTGAAGAT |
| SAG-RELATED SEQUENCE SRS23 | HHA_239090 | qpcr | TGGACCCTAAGGACGTACAA | GATCCATCGACAAGGGAAGTG |
| AP2 DOMAIN TRANSCRIPTION FACTOR AP2IV-4 | HHA_318470 | qpcr | CTGGAGAGCGAAGGGAATG | GACTGGAGAGGGAGAATGAAAG |
| AP2 DOMAIN TRANSCRIPTION FACTOR AP2VI-1 | HHA_240460 | qpcr | CACTGAGGCCTATTCAGAGATG | CATGACTTTCCGGGTTTCTTTG |
| CALCIUM-DEPENDENT PROTEIN KINASE (CDPK1) | HHA_301440 | qpcr | ACTGACAGCCATCTTCCATAAG | ACTGGAATCTTGACCCTTCATC |
| BRADYZOITE ANTIGEN BAG1 | HHA_259020 | qpcr | CTCGAAAGAGTCGGAGAAAGTG | GATTCCGTCGGGCTTGTAAT |
| lactate dehydrogenase LDH2 | HHA_291040 | qpcr | GCCAGCCCTTTCTGCTATAA | CGTATCGTGAAGCCCATACTC |
| ENOLASE 1 ENO1 | HHA_268860 | qpcr | GACCAAGAATGCATCCAAACAC | GAAAGGGTCTTCGACCGATATG |
| SAG-RELATED SEQUENCE SRS27B | TGVEG_258810 | qpcr | CGTAAGGGAGGATCAGAGAATG | CCATCCGACTTCTTGCATTTG |
| RHOPTRY PROTEIN ROP18 | TGVEG_205250 | qpcr | GACGGCATCTGGGACTATTT | GTTTCTGAGGCTCTCGATTCA |
| WD DOMAIN, G-BETA REPEAT-CONTAINING PROTEIN | TGVEG_219450 | qpcr | ACGGGCCTAAGACACAATTC | ACGCCAAGAGAACTCGAAAG |
| HYPOTHETICAL PROTEIN_1 | TGVEG_253150 | qpcr | ATTGGCACTCAGGGTCTTTC | CCTGCCTTCTTGAGCTTCTT |
| PUTATIVE TRANSMEMBRANE PROTEIN | TGVEG_215328 | qpcr | CCGAGACAACGAGGATGAATAC | CGTCAGTACTCGAGGAGAAGTA |
| HYPOTHETICAL PROTEIN_3 | TGVEG_278680 | qpcr | CACACAGCGCCTAGATAGTTT | AGAGGATGATTTGGCTGAAGG |
| SAG-RELATED SEQUENCE SRS23 | TGVEG_239090 | qpcr | GCTCTCACTTCCCTTGTTGAT | CGGCTGGGAGTTCACTTATAC |
| AP2 DOMAIN TRANSCRIPTION FACTOR AP2IV-4 | TGVEG_318470 | qpcr | CTGGAGAGCGAAGGGAATG | GACTGGAGAGGGAGAATGAAAG |
| AP2 DOMAIN TRANSCRIPTION FACTOR AP2VI-1 | TGVEG_240460 | qpcr | CTCAACTAACCACAGACCCATAG | CACTTCCAAACTTCCGCTATCT |
| CALCIUM-DEPENDENT PROTEIN KINASE (CDPK1) | TGVEG_301440 | qpcr | GACTCCGACAACTCAGGAAAG | GACTTCGCCGTCGTTATTCT |
| BRADYZOITE ANTIGEN BAG1 | TGVEG_259020 | qpcr | GAGCGAGTGTCCGGTTATTT | AGAACGCCGTTGTCCATT |
| lactate dehydrogenase LDH2 | TGVEG_291040 | qpcr | GTACTATGCTCCAGCCCTTTC | CCATACTCACCCTGGCAATAC |
| ENOLASE 1 ENO1 | TGVEG_268860 | qpcr | CCCATAGATCAGGTGAAACAGAG | GGACTCCTCAATCCGCATAAG |
| UPRT_dsred_cassette, for CRISPR and pcr screen | N/A | dsRED Repair Template with UPRT-targeting flanks | AGTTTCCTTTTACTCCAAGATCTGTATAGCGTGGTACTCGTCACGAA | CAAGCCGCTTTCCATCGACTCGCCAGTCGACTGGAACTACGGTGTTTG |
| Hham34F, Hham3R (Hhspecific) | N/A | Distinguish *T. gondii* from *H. hammondi*, amplifies 234bp fragment of Hh | ATCCCATTCCGGCTTCAGTCTTTC | ACAGCGGAGCCGAAGTTGGTTT |
| dsRED Scrn 1 | N/A | Amplifies 75bp fragment of dsRED | AGTTCCAGTACGGCTCCAAG | GGGGAAGGACAGCTTCTTGT |
| dsRED Scrn 2 | N/A | Amplifies 391bp fragment of dsRED | AGTTCCAGTACGGCTCCAAG | TAGTAGTAGCCGGGCAGCTT |
