# Supplementary material for: Dissection of the in vitro developmental program of Hammondia hammondi reveals a link between stress sensitivity and life cycle flexibility in Toxoplasma gondii
Source: eLife. 2018 May 22;7:e36491. doi: 10.7554/eLife.36491 (PMC5963921; doi:10.7554/eLife.36491)
Supplement: Transparent reporting form [file elife-36491-transrepform.docx]

***eLife’s* transparent reporting form**

We encourage authors to provide detailed information *within their submission* to facilitate the interpretation and replication of experiments. Authors can upload supporting documentation to indicate the use of appropriate reporting guidelines for health-related research (see [EQUATOR Network](http://www.equator-network.org/%20)), life science research (see the [BioSharing Information Resource](https://biosharing.org/)), or the [ARRIVE guidelines](http://www.plosbiology.org/article/info:doi/10.1371/journal.pbio.1000412) for reporting work involving animal research. Where applicable, authors should refer to any relevant reporting standards documents in this form.

If you have any questions, please consult our Journal Policies and/or contact us: [editorial@elifesciences.org](mailto:editorial@elifesciences.org).

**Sample-size estimation**

- You should state whether an appropriate sample size was computed when the study was being designed
- You should state the statistical method of sample size computation and any required assumptions
- If no explicit power analysis was used, you should describe how you decided what sample (replicate) size (number) to use

Please outline where this information can be found within the submission (e.g., sections or figure legends), or explain why this information doesn’t apply to your submission:

We did not conduct any statistical methods of sample size computation in this

study. Due to the difficulty associated with generating oocysts, we based all of

our sample sizes on oocyst availably during the time of experimentation.

Sample size for data in Figure 1 can be found in

the methods sections entitled “Quantification of sporozoite viability and replication rate,”(Line 556), and within the figure legend.

Sample size for data in Figure 2 can be found in the methods sections entitled “Quantification of spontaneous *Dolichos biflorus*-positive cyst formation *in vitro,”* (Line 578).This information can also be found in the legends for these figures.

For Figure 3 and 8, sample size information can be found in the methods

section entitled, “RNA sequencing and data processing,”(Line 740). The sample size for Figure 3- Figure supplement 1 are the same. For the data presented in Figure 3-Figure supplement 2, 3 replicates the sample size is 1. For Figure 3-Figure supplement 3, sample size information can be found in the methods section entitled, “cDNA synthesis and qPCR,” (Line 826) and in the legend.

For Figure 4, the sample size was limited to 1 due to oocyst availability.

For Figure 5 sample size information can be found in the figure legend.

For Figure 6, the sample size is 1.

For Figure 7, sample size information can be found in the results section entitled, “Induction of bradyzoite formation in *T. gondii* and *H. hammondi*,” (Line 605). The 48 hour pH treatment experiment was conducted twice (2 biological replicates) while the 72 hour and 96 hour induction experiment was conducted once in total.

For Figure 8, (and associated supplemental figure) the experiment was completed once in total with three replicates for each treatment.

**Replicates**

- You should report how often each experiment was performed
- You should include a definition of biological versus technical replication
- The data obtained should be provided and sufficient information should be provided to indicate the number of independent biological and/or technical replicates
- If you encountered any outliers, you should describe how these were handled
- Criteria for exclusion/inclusion of data should be clearly stated
- High-throughput sequence data should be uploaded before submission, with a private link for reviewers provided (these are available from both GEO and ArrayExpress)

Please outline where this information can be found within the submission (e.g., sections or figure legends), or explain why this information doesn’t apply to your submission:

For experiments described in this paper, we define technical replicate as an

infection of a distinct unit (host cells grown separately) initiated from

sporozoites derived from the same *in vitro* excystation. We define a biological

replicate as infections elicited sporozoites obtained from different *in vitro*

excystation.

For Figure 1, replicate information can be found in the material and methods section entitled “Quantification of sporozoite viability and replication rate,”(Line 556), and within the figure legend. Three technical replicates were performed for a total of 2 experiments or biological replicates.

For Figure 2, replicate information can be found in the materials and

methods section entitled, “Quantification of spontaneous *Dolichos biflorus* positive cyst formation *in vitro*,” (Line 578). For A and B, three technical replicates were performed. For C and D, 1 replicate was quantified, however we see similar results in additional analyses.

For Figure 3 and associated supplemental figures, replicate information can be found in the methods section entitled, “RNA sequencing and data processing,” (Line 740) and in the figure legend. RNAseq was performed in technical triplicate, except for the D4 samples due to parasite availability.

For Figure 3-Figure Supplement 3, sample size information can be found in the

methods section entitled, “cDNA synthesis and qPCR,” (Line 826) and in the legend.

The data from the experiment described in Figure 4 was performed once in its

entirety, however the phenomenon of subculture was repeated in order to

perform the experiments described in Figures 5 and 6.

Figures 5 and 6 were performed once in their entirety. For each experiment, 2 mice were used and the infection from each mouse was analyzed as a technical replicate. The experiment described in 5B was conducted twice (2 biological replicates) and similar results were observed the second time.

For Figure 7, 2-3 technical replicates were performed for each treatment for each time point. The 48 hour treatment was conducted twice (2 biological replicates).

For Figure 8, RNAseq and qPCR were performed in technical triplicate for all conditions.

**Statistical reporting**

- Statistical analysis methods should be described and justified
- Raw data should be presented in figures whenever informative to do so (typically when N per group is less than 10)
- For each experiment, you should identify the statistical tests used, exact values of N, definitions of center, methods of multiple test correction, and dispersion and precision measures (e.g., mean, median, SD, SEM, confidence intervals; and, for the major substantive results, a measure of effect size (e.g., Pearson's r, Cohen's d)
- Report exact p-values wherever possible alongside the summary statistics and 95% confidence intervals. These should be reported for all key questions and not only when the p-value is less than 0.05.

Please outline where this information can be found within the submission (e.g., sections or figure legends), or explain why this information doesn’t apply to your submission:

Detailed information with regards to all statistical analyses performed can be

found in the legends for each figure and also in the results text.

(For large datasets, or papers with a very large number of statistical tests, you may upload a single table file with tests, Ns, etc., with reference to sections in the manuscript.)

**Group allocation**

- Indicate how samples were allocated into experimental groups (in the case of clinical studies, please specify allocation to treatment method); if randomization was used, please also state if restricted randomization was applied
- Indicate if masking was used during group allocation, data collection and/or data analysis

Please outline where this information can be found within the submission (e.g., sections or figure legends), or explain why this information doesn’t apply to your submission:

**Additional data files (“source data”)**

- We encourage you to upload relevant additional data files, such as numerical data that are represented as a graph in a figure, or as a summary table
- Where provided, these should be in the most useful format, and they can be uploaded as “Source data” files linked to a main figure or table
- Include model definition files including the full list of parameters used
- Include code used for data analysis (e.g., R, MatLab)
- Avoid stating that data files are “available upon request”

Please indicate the figures or tables for which source data files have been provided:

Five supplemental files are being provided. File Supplement 1 includes the D4D5 HFF RNAseq data and the 24hour THP-2 RNAseq data. Raw reads, Log_2_FPM values, and statistical analysis from DESeq2 are all included. File supplement 2 included the pH vs control RNAseq data with the same values described above. File Supplement 3 includes all of the primers used in this study and a description of how they were used. File Supplements 4 and 5 indicate which genes we identified from all of the gene sets used in GSEA analysis for the spontaneous D4/D15/24H RNAseq experiments and the pH vs control RNAseq experiments, respectively.
